# Supplementary material for: Self-Healing and Super-Elastomeric PolyMEA-co-SMA Nanocomposites Crosslinked by Clay Platelets
Source: Gels. 2022 Oct 15;8(10):657. doi: 10.3390/gels8100657 (PMC9601507; doi:10.3390/gels8100657)
Supplement: Supplementary file 1 [file gels-08-00657-s001.zip › gels-1957668-supplementary.pdf]

# SUPPLEMENTARY INFORMATION

# Self-healing and super-elastomeric PolyMEA-co-SMA nanocomposites crosslinked by clay platelets

Beata Strachota[1], Adam Strachota[1*], Katarzyna Byś[1], Ewa Pavlova[1], Jiří Hodan[1], Beata Mossety-Leszczak[2]

[1] *Institute of Macromolecular Chemistry, Czech Academy of Sciences Heyrovskeho nam. 2, CZ-162 00 Praha, Czech Republic*
[2] *Faculty of Chemistry, Rzeszow University of Technology, al. PowstancowWarszawy 6, PL-35-959 Rzeszow, Poland*

# 1 Morphology: TEM

2R

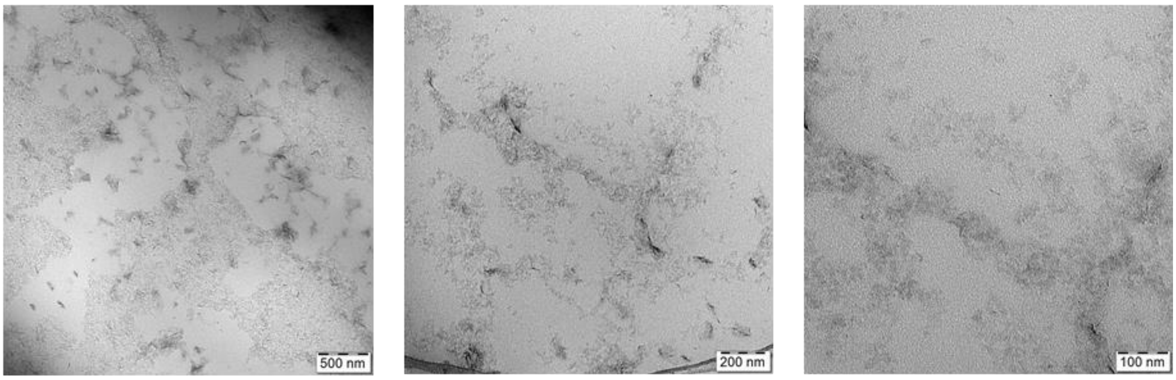


2R-10S

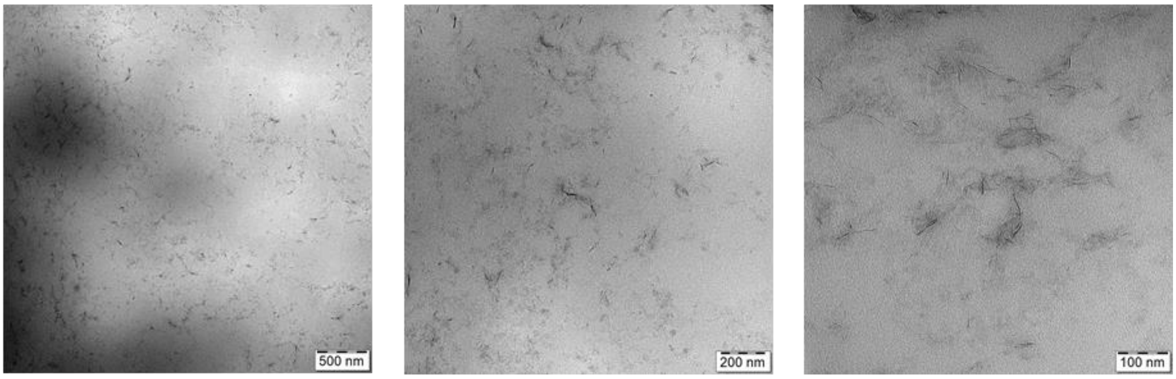


***SI-Fig. S1.*** *Morphology of the nanocomposites "2R" (top row) and 2R-10S (bottom row), displayed at different zoom: scalebar: 500 nm (left), 200 nm (centre), and 100 nm (right).*

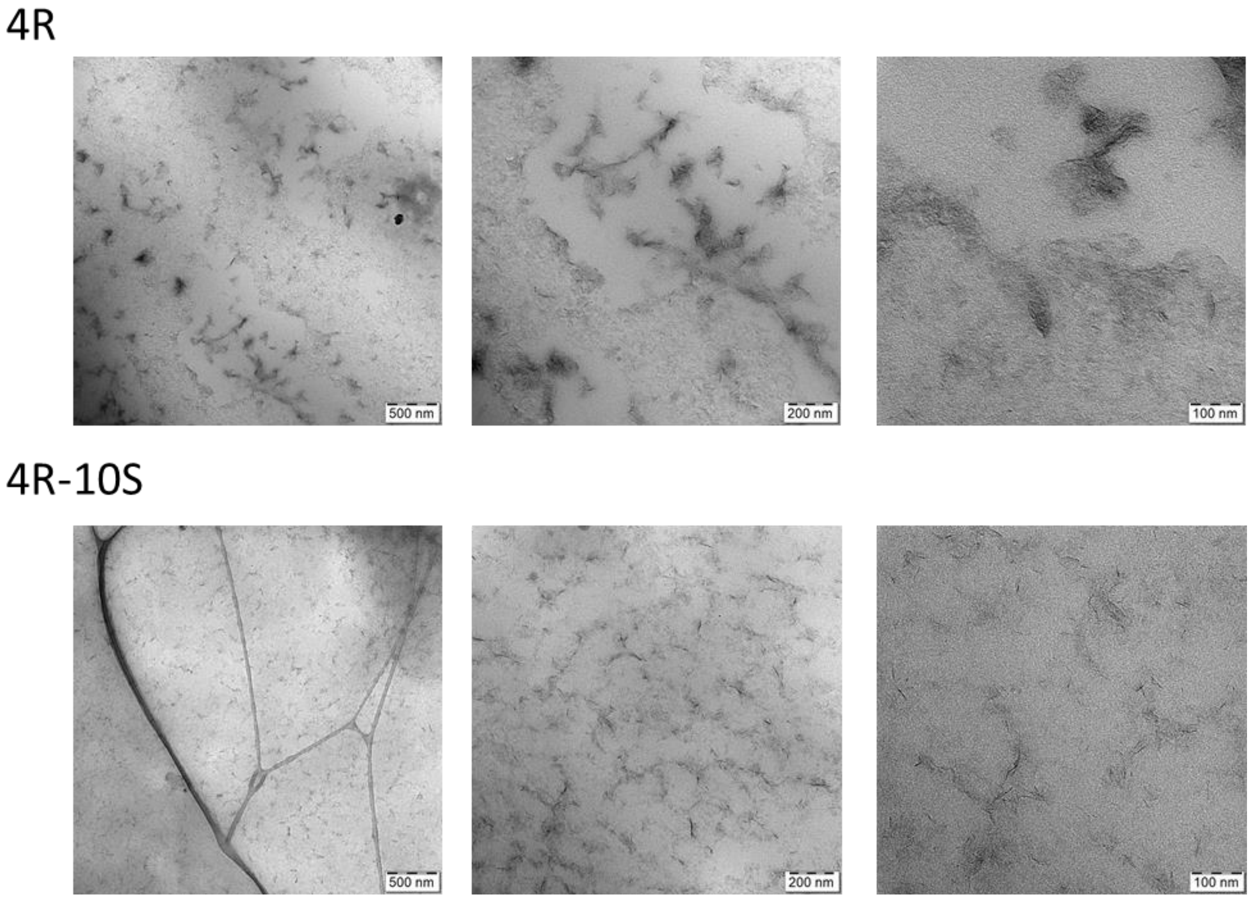


***SI-Fig. S2.*** *Morphology of the nanocomposites "4R" (top row) and 4R-10S* (bottom row), *displayed at different zoom: scalebar: 500 nm (left), 200 nm (centre), and 100 nm (right).*

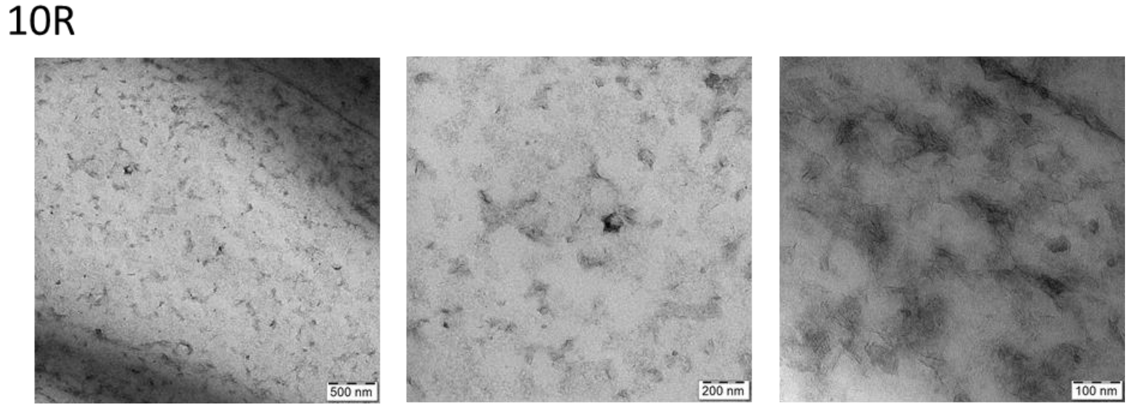


***SI-Fig. S3.*** *Morphology of the nanocomposite "10R" displayed* at different zoom: scalebar: *500 nm (left), 200 nm (centre), and 100 nm (right).*

# 2 DMTA: Thermo-mechanical properties

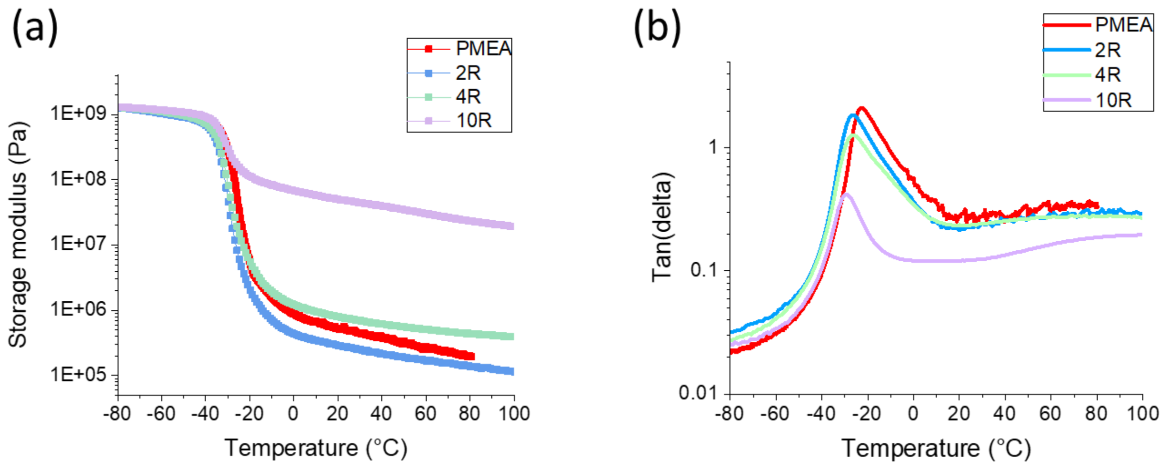


***SI-Fig. S4.*** *Effect of clay (RDS, acting as physical crosslinker) on thermo-mechanical properties: DMTA analysis of neat polyMEA and of nanocomposites with 2, 4, and 10 wt.% of RDS clay (PMEA, 2R, 4R and 10R, respectively) : (a) curves of the temperature-dependence of storage shear moduli (G'); (b) curves of the temperature-dependence of the loss factor ( tan(delta) ).*

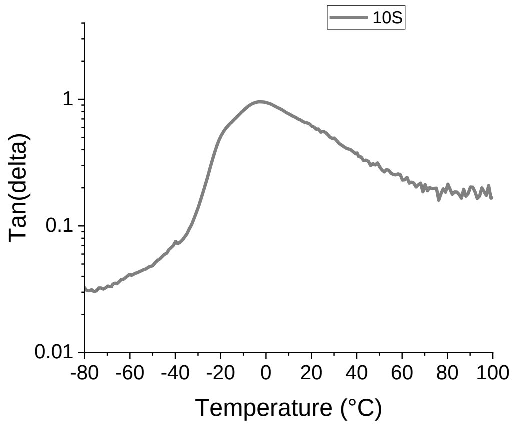


***SI-Fig. S5.*** *DMTA analysis of the SMA-free 'linear' co-polymer poly(MEA-co-10mol%SMA): curve of the temperature-dependence of the loss factor tan(delta).*

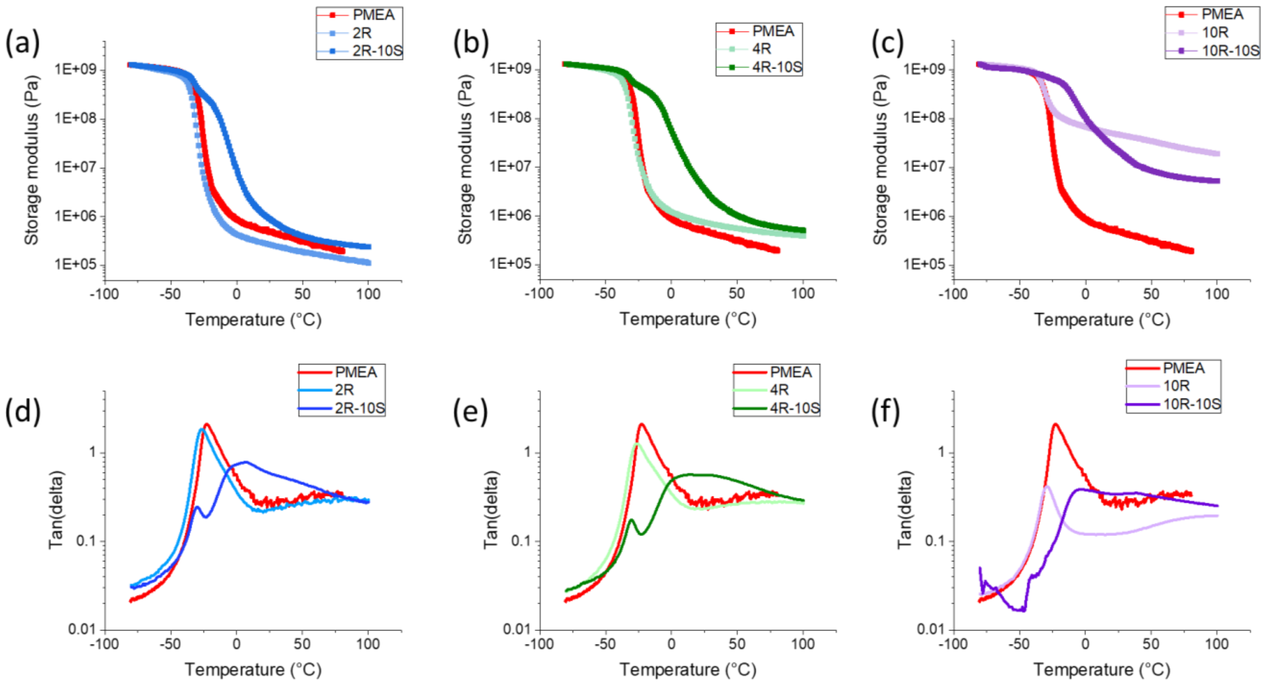


***SI-Fig. S6.*** *Dynamic-mechanical thermal analysis (DMTA) of nanocomposites loaded with 2 (a, d), 4 (b, e) and 10 wt.% of clay (c, f): in each graph, the SMA-free composite is compared with its analogue doped by 10 mol% of SMA and with the neat polyMEA matrix; the top row (a – c) compares curves of temperature-dependent storage shear moduli (G'); the bottom (d – f) row compares the curves of the temperature-dependence of the loss factor tan(delta).*

# 3 DSC: Thermal transitions

(a)

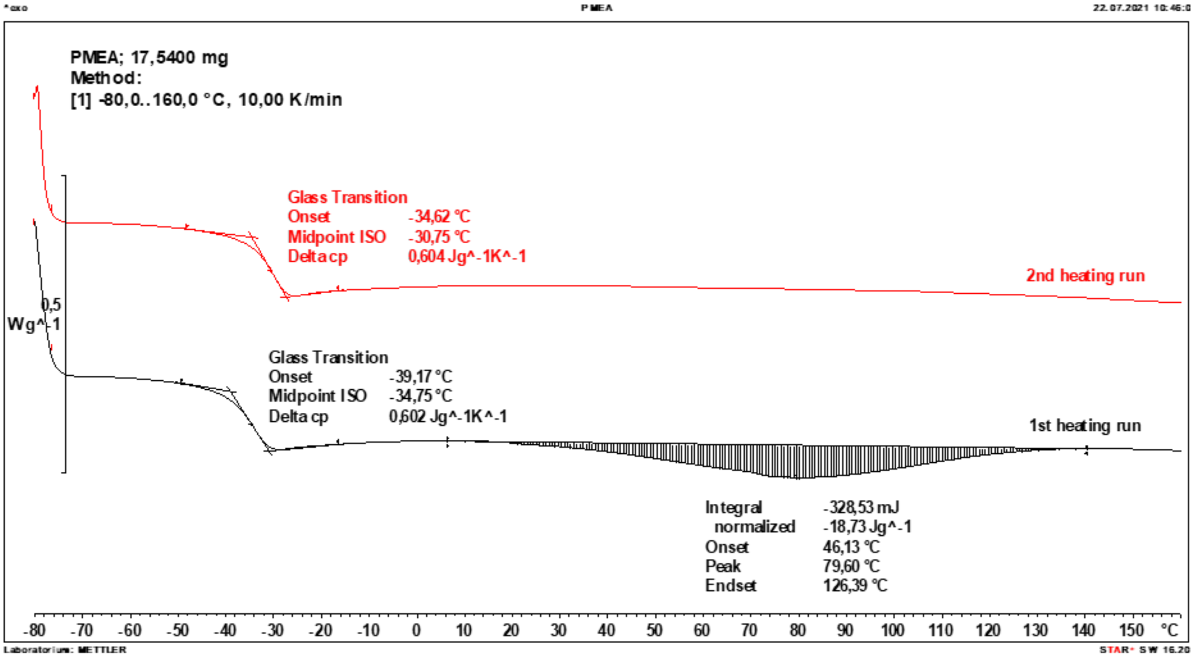


(b)

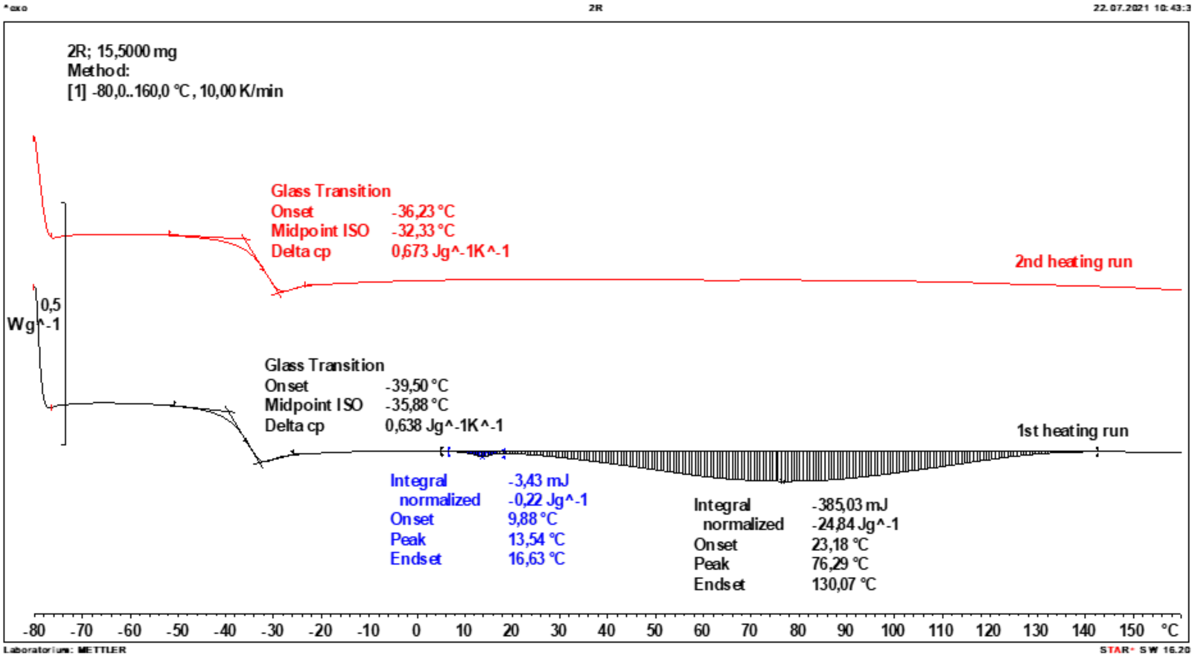


***SI-Fig. S7. (part 1/3):*** *DSC traces of (a) neat polyMEA; and (b) of the SMA-free nanocomposite 2R, based on polyMEA filled with 2 wt.% of RDS clay.*

(c)

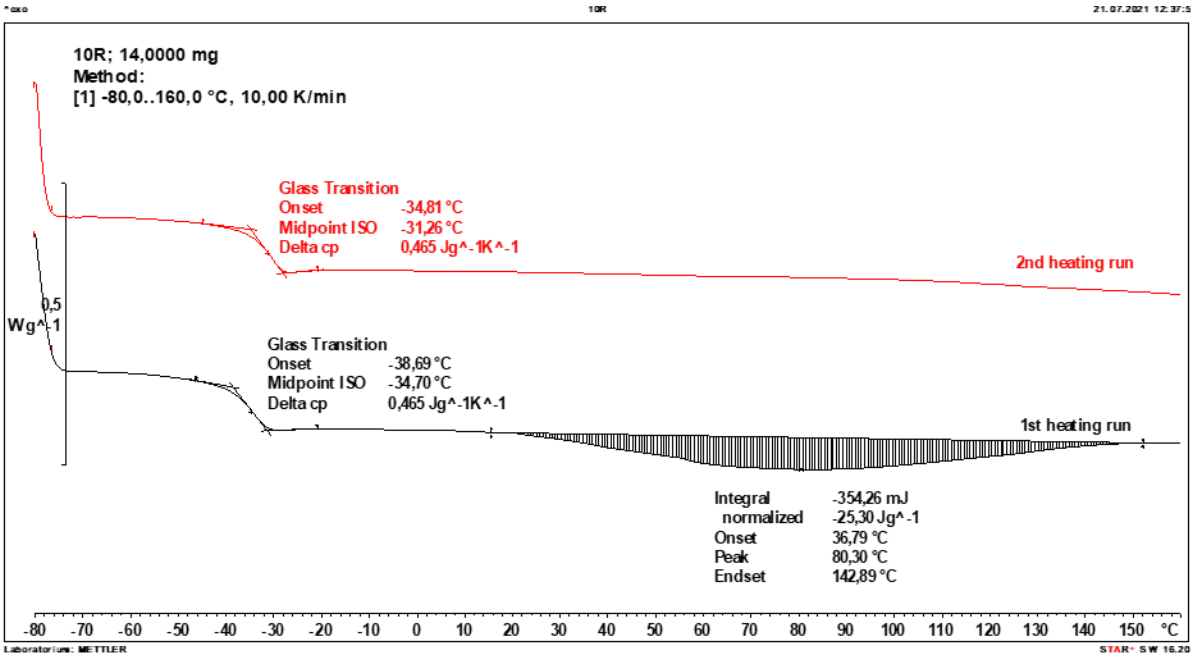


(d)

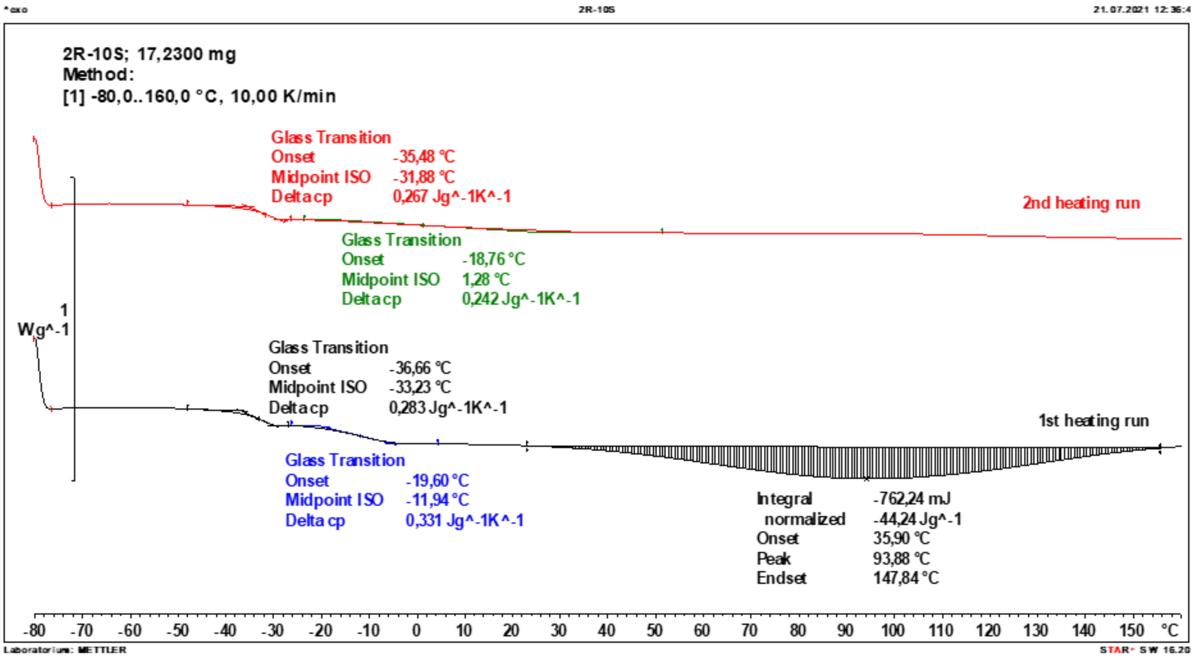


***SI-Fig. S7. (part 2/3):*** *DSC traces of (c) the SMA-free nanocomposite 10R, based on polyMEA filled with 10 wt.% of RDS clay; and (d) of the nanocomposite 2R-10S filled with 2% of clay and doped with 10 mol% of SMA.*

(e)

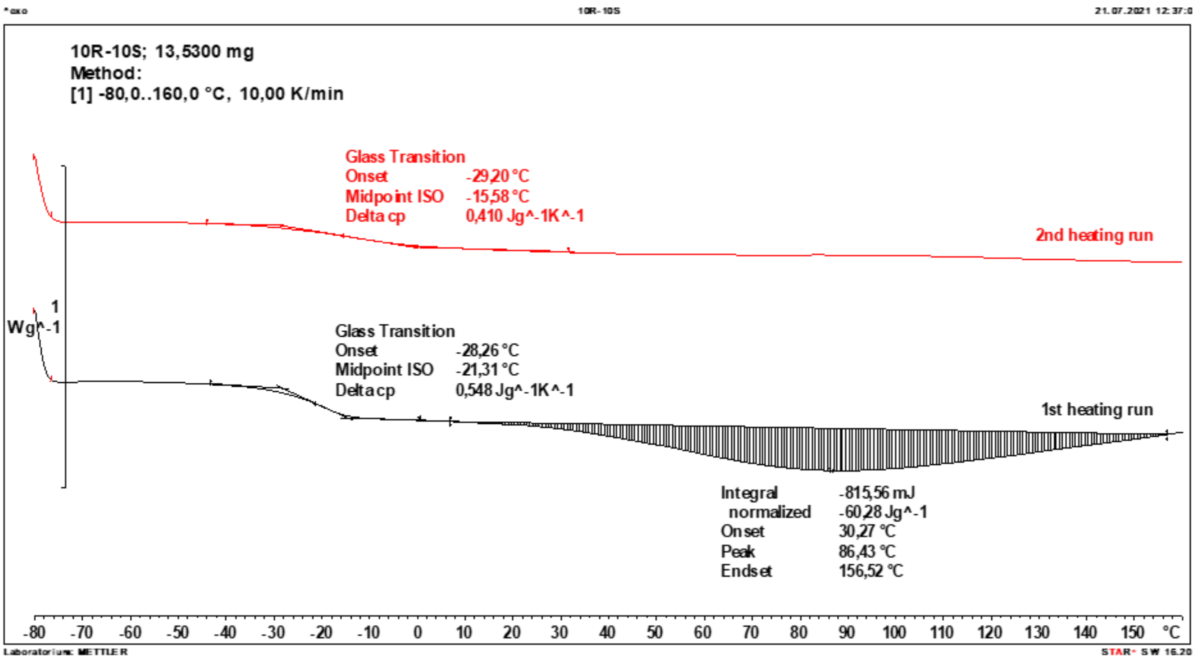


***SI-Fig. S7. (part 3/3):*** *DSC trace of (e) the nanocomposite 10R-10S, based on polyMEA filled with 10 wt.% of RDS clay and doped with 10 mol% of SMA.*

# 4 Self-healing of disrupted samples

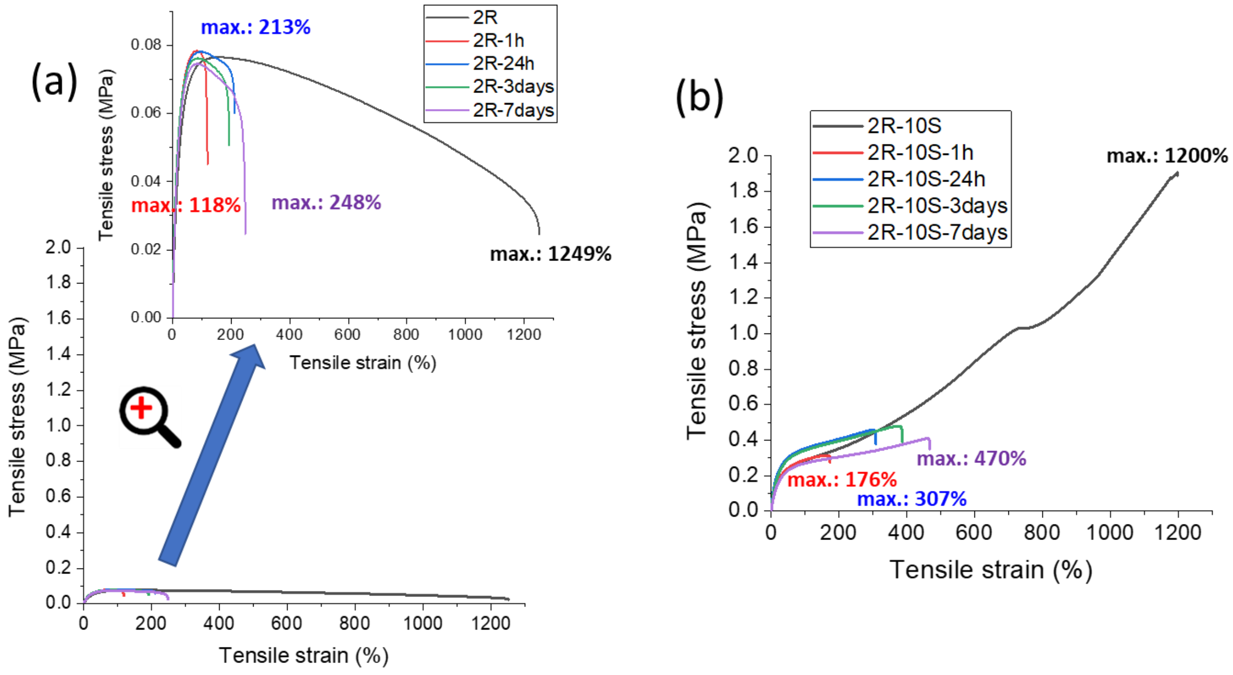


***SI-Fig. S8.*** *Self-healing of nanocomposite elastomers based on poly(MEA) crosslinked by 2 wt.% of clay: (a) SMA-free ("2R") and (b) SMA-doped (10 mol%) system ("2R-10S").*

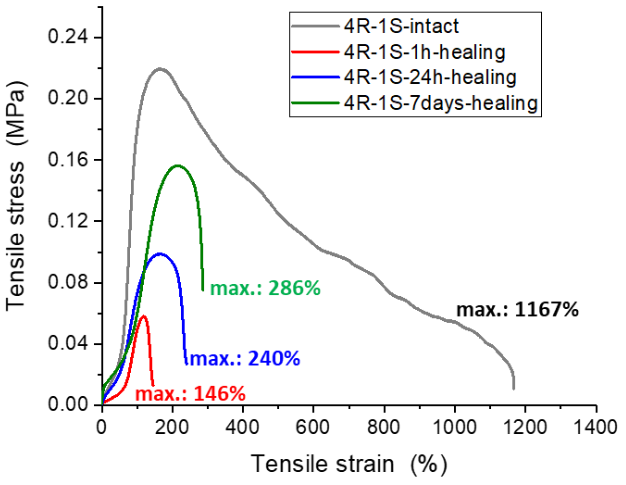


***SI-Fig. S9.*** *Self-healing of 4R-1S, a nanocomposite elastomer based on poly(MEA) crosslinked by 4 wt.% of clay and doped by 1 mol% of SMA.*

# 5 Creep vs. elasticity: Cyclic stretching tests

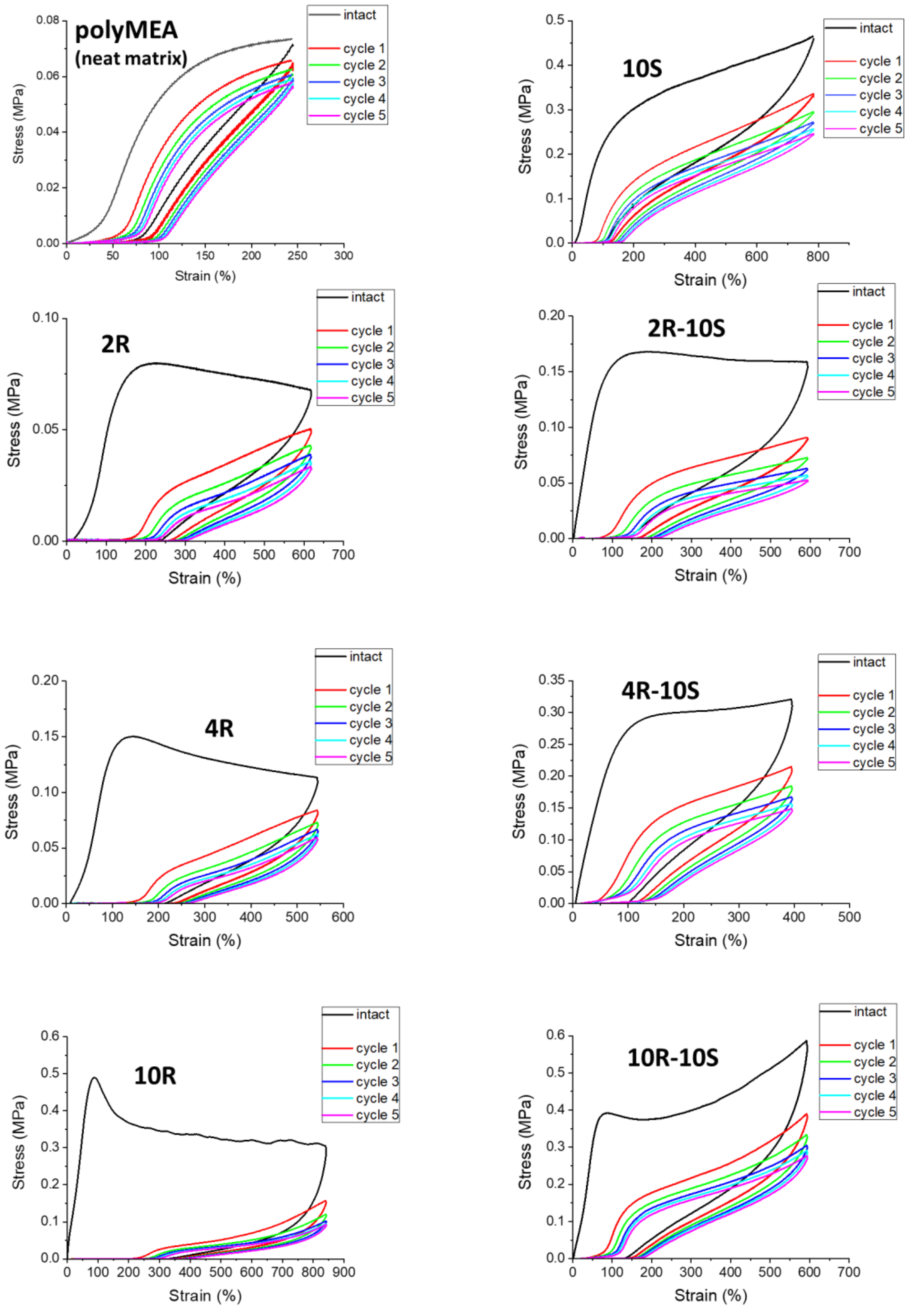


***SI-Fig. S10.*** *Repeated cyclic loading test for (left column) the 'linear' polymer (polyMEA) and for the SMA-free nanocomposites 2R, 4R and 10R; and (right column) for their analogues doped by 10 mol% of SMA: 10S (= doped 'linear' polyMEA), 2R-10S, 4R-10S and 10R-10S; the graphs are maximally zoomed and have different X and Y scales; the comparison shows the different nature of response to cyclic loading.*

# 6 Self-recovery: internal self-healing

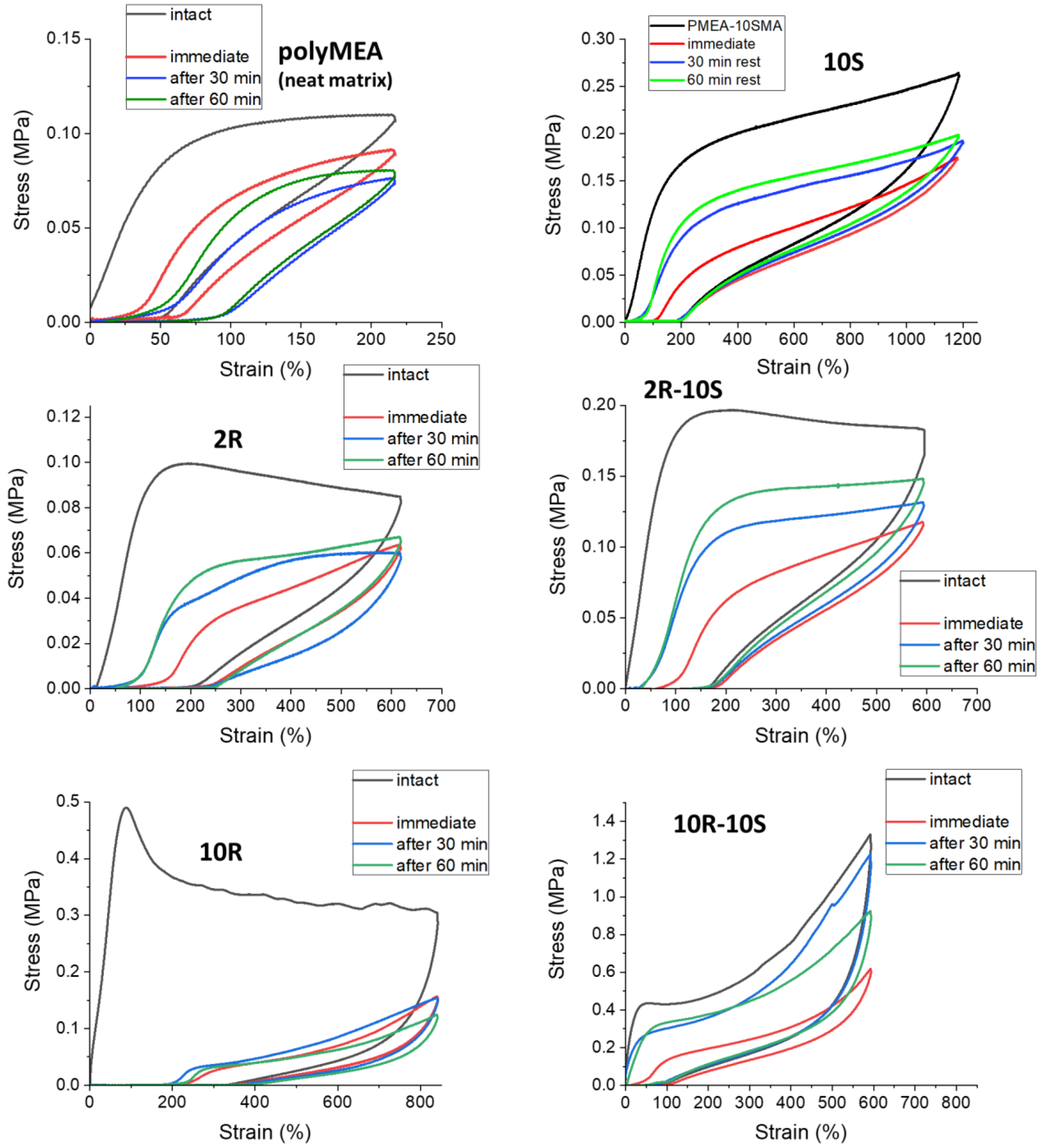


***SI-Fig. S11.*** *Images of self-recovery tests of clay-free samples polyMEA and 10S (= doped 'linear' polyMEA doped with 10 mol% of SMA), of the SMA-free nanocomposites 2R and 10R, and of their SMA-doped analogues 2R-10S and 10R-10S.*
